# Modeling Dengue Vector Dynamics under Imperfect Detection: Three Years of Site-Occupancy by *Aedes aegypti* and *Aedes albopictus* in Urban Amazonia

**DOI:** 10.1371/journal.pone.0058420

**Published:** 2013-03-05

**Authors:** Samael D. Padilla-Torres, Gonçalo Ferraz, Sergio L. B. Luz, Elvira Zamora-Perea, Fernando Abad-Franch

**Affiliations:** 1 Graduate Program in Ecology, Instituto Nacional de Pesquisas da Amazônia, Manaus, Amazonas, Brazil; 2 Biological Dynamics of Forest Fragments Project, Smithsonian Tropical Research Institute/Instituto Nacional de Pesquisas da Amazônia, Manaus, Amazonas, Brazil; 3 Instituto Leônidas e Maria Deane – Fiocruz Amazônia, Manaus, Amazonas, Brazil; University of Texas Medical Branch, United States of America

## Abstract

*Aedes aegypti* and *Ae. albopictus* are the vectors of dengue, the most important arboviral disease of humans. To date, *Aedes* ecology studies have assumed that the vectors are truly absent from sites where they are not detected; since no perfect detection method exists, this assumption is questionable. Imperfect detection may bias estimates of key vector surveillance/control parameters, including site-occupancy (infestation) rates and control intervention effects. We used a modeling approach that explicitly accounts for imperfect detection and a 38-month, 55-site detection/non-detection dataset to quantify the effects of municipality/state control interventions on *Aedes* site-occupancy dynamics, considering meteorological and dwelling-level covariates. *Ae. aegypti* site-occupancy estimates (mean 0.91; range 0.79–0.97) were much higher than reported by routine surveillance based on ‘rapid larval surveys’ (0.03; 0.02–0.11) and moderately higher than directly ascertained with oviposition traps (0.68; 0.50–0.91). Regular control campaigns based on breeding-site elimination had no measurable effects on the probabilities of dwelling infestation by dengue vectors. Site-occupancy fluctuated seasonally, mainly due to the negative effects of high maximum (*Ae. aegypti*) and minimum (*Ae. albopictus*) summer temperatures (June-September). Rainfall and dwelling-level covariates were poor predictors of occupancy. The marked contrast between our estimates of adult vector presence and the results from ‘rapid larval surveys’ suggests, together with the lack of effect of local control campaigns on infestation, that many *Aedes* breeding sites were overlooked by vector control agents in our study setting. Better sampling strategies are urgently needed, particularly for the reliable assessment of infestation rates in the context of control program management. The approach we present here, combining oviposition traps and site-occupancy models, could greatly contribute to that crucial aim.

## Introduction

Dengue is the most common arboviral disease of humans [1]–[3]. About 50 million people contract dengue annually, and an estimated 22,000 die from severe forms of the disease [3], [4]. Dengue virus is transmitted by mosquitoes of the genus *Aedes*, particularly *Aedes aegypti* and *Ae. albopictus* [5]. In the absence of effective drugs or vaccines, prevention of dengue infections and severe dengue forms heavily relies upon vector control. However, despite massive spending and some encouraging results (e.g., [6]–[9]), neither vector populations nor, consequently, dengue transmission are currently under control; on the contrary, they are both clearly expanding [2], [10]. In South America, dengue incidence increased from ∼16/100,000 population in the 1980s to ∼72/100,000 in 2000–2007 [11].

*Aedes aegypti*, a species native to Africa, has successfully adapted to urban environments around the world; it preferentially breeds in artificial containers (where desiccated eggs can remain viable for months), rests within houses, and feeds on human blood [12], [13]. These traits have favored its man-mediated dispersal throughout the tropics [14], [15], and, together with its capacity to transmit dengue virus, have transformed *Ae. aegypti* in a major public health concern [16]. *Ae. albopictus* is more eclectic: it exploits both urban and rural tropical-subtropical habitats, makes use of natural and artificial breeding sites, and feeds on either humans or non-human vertebrates [17], [18]; this species, however, is less efficient than *Ae. aegypti* at transmitting dengue virus [18].

Dengue vector control is largely based on a combination of strategies aimed at eliminating *Aedes* breeding sites (either physically or by means of larvicides) and reducing adult mosquito populations (through environmental insecticide application) [6]–[9]. The design, implementation, and assessment of such strategies require detailed knowledge of vector population ecology, including the estimation of dwelling infestation rates [19], [20]. In general, vector control interventions are expected to have a negative effect on infestation by *Ae. aegypti* and *Ae. albopictus* at the local scale. Measuring such an effect requires reliable methods for ascertaining infestation; yet, detection of most animal species, including disease vectors, is rarely, if ever, perfect [21], [22]. Here, we treat infestation as the probability that a dwelling is occupied by vectors (i.e. site-occupancy) and use a hierarchical modeling approach to analyze the dynamics of site-occupancy by *Ae. aegypti* and *Ae. albopictus*. Our analysis is based on three years of oviposition trap (ovitrap) data from a central-Amazon urban setting. Taking imperfect detection into account, we quantify the effects of routine control interventions and selected environmental variables on the main indicator used in vector control program management – dwelling infestation rates – and on its temporal change.

## Materials and Methods

### Study Setting

**Figure 1:**
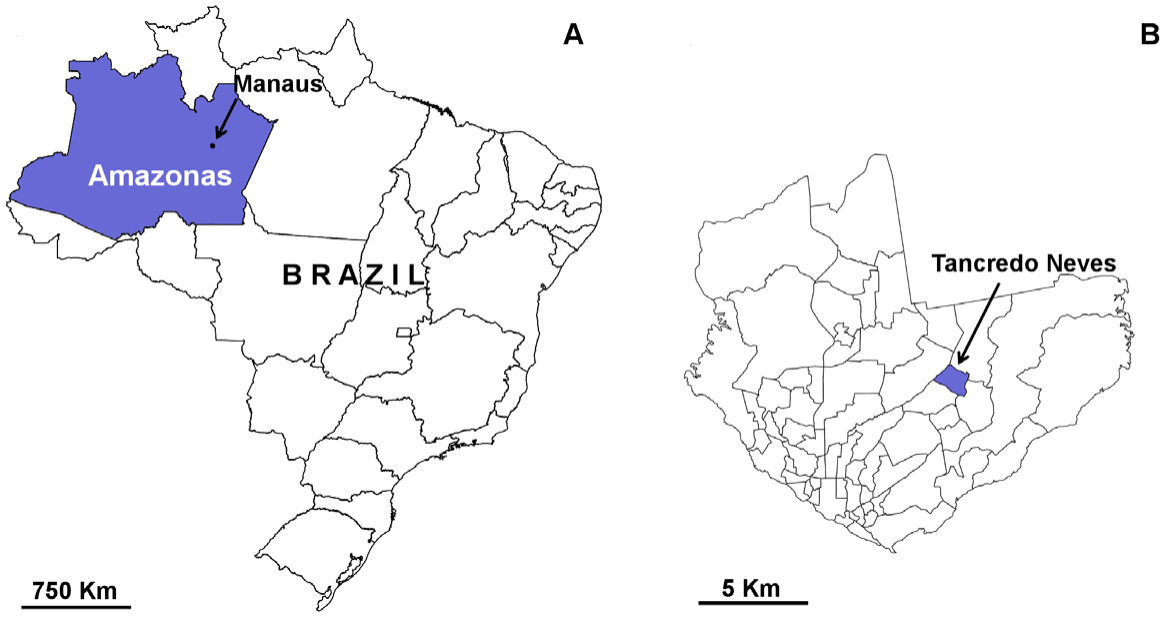
Study area. Manaus, state of Amazonas, Brazil (A) and Tancredo Neves neighborhood (B).

**Figure 2:**
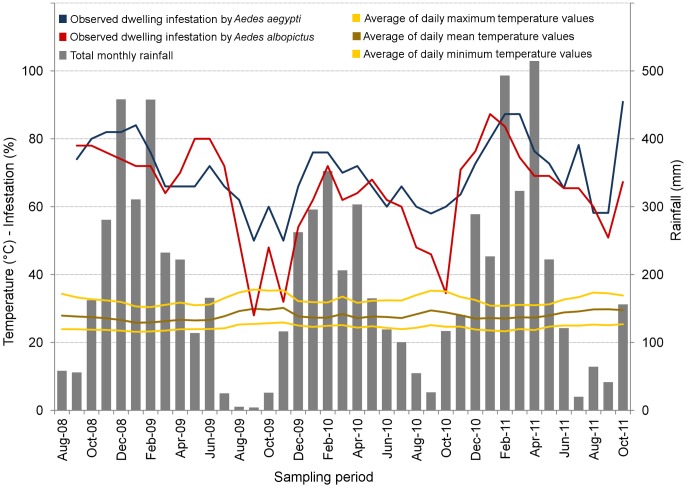
Observed dwelling infestation and meteorological variables during the study period. Dwelling infestation (%; left *y* axis) by *Aedes aegypti* and *Ae. albopictus*; total monthly rainfall (mm; right *y* axis); and monthly averages of daily mean, minimum, and maximum temperatures (°C; left *y* axis).

With a population of about 1.8 million, Manaus (3°6′S, 60°1′W) is the largest urban center of the Amazon basin (Figure 1). The city lies on the north bank of the Negro river and is surrounded by rainforest. The climate is tropical, warm and humid, with a relatively strong seasonality of rainfall and, to a lesser extent, temperature (Figure 2). After being declared eradicated from Brazil in the 1950s [15], *Ae. aegypti* reinfested Manaus in the late 1990s [23] and is currently widespread across all its neighborhoods [24]. *Ae. albopictus* was first recorded in 2002 [25], and is now also widespread [24]. Dengue transmission is endemic (i.e., occurs continuously) in the city, with recurrent epidemics and records of all known dengue virus serotypes [26]. As in other settings, dengue control in Manaus relies on dwelling visits by municipal or state agents, who physically eliminate breeding sites or treat them with larvicides; in “emergency” situations (in practice, when dengue cases begin to soar), environmental insecticide spraying aimed at reducing adult mosquito density is also used [27]. Vector control agencies also conduct regular infestation surveys on a random sample of dwellings in each neighborhood (see details in ref. [28]). The results of these ‘rapid larval surveys’ are used to set priorities and make decisions about control interventions, with control teams usually deployed to a neighborhood when dwelling infestation rises above 2%; officially, the Brazilian control program aims to keep dwelling infestation below 1% [20].

### Sampling Strategy

We selected an area of ∼250,000 m^2^ within the Manaus neighborhood of Tancredo Neves (Figure 1) for vector monitoring. This neighborhood is frequently infested by both target mosquito species, and about 1500 dengue cases were notified during the study period (refs. [24], [29], [30] and unpublished Municipal Health Department data). The typical Tancredo Neves dwelling – our unit of occupancy analysis – consists of a brick-walled house with a courtyard in a ∼10×20 m plot. In 2008, we randomly selected 50 dwellings for monthly sampling, and in 2010 we added five more dwellings, which were also sampled on a monthly basis; all residents gave informed consent. During the first 25 months, we used a combination of 2–4 ovitraps and 0–2 Adultraps [31]; afterwards, only the more sensitive ovitraps (3 per dwelling and month) were used. Traps contained hay infusion [32] and were operated for six days/month. In total, our analyses use data from nearly 5800 trap-weeks. Each month, mosquito larvae were identified to species, with the result of each individual trap recorded separately. Thus, for each dwelling and month between September 2008 and October 2011, we have a ‘detection history’ consisting of a series of binary outcomes (present = 1 and absent = 0), with one outcome per trap and per mosquito species. These ‘detection histories’ are central to our approach: by repeatedly sampling each dwelling in each month and recording each trap’s result separately, we can derive an estimate of the rate of false-negative trap results and use it to correct infestation estimates (see below). This summarizes the hierarchical nature of our models – detection histories result from the interplay of two hierarchically juxtaposed processes, where the sampling process of detecting a species in one trap is conditioned on the biological process of dwelling occupancy by that species. The use of multiple traps per dwelling enables formal separation of the two processes.

### Covariates

In order to model the relation between environmental variables and infestation we obtained daily data on total rainfall, as well as on maximum, mean, and minimum temperature, from the Brazilian National Meteorological Institute (INMET). We chose these environmental metrics, or covariates, because we considered them potentially relevant for the spatial-temporal distribution of our two target species [33]–[36]. Because we had no prior information on possible time lags between meteorological changes and their effects on local mosquito populations, we decided to relate meteorological information and each month’s occupancy in three different ways: (i) looking at meteorological covariates measured, for each month, during sampling and the previous week (denoted *1-week-lag* below); (ii) looking at covariates measured during sampling and the previous two weeks (*2-week-lag*); and (iii) looking at covariates measured during the four weeks before sampling (*4-week-lag*). All meteorological measurements were standardized to mean zero and standard deviation one before analysis.

Apart from rainfall and temperature, we also registered dwelling-level traits throughout the last 13 months of monitoring. Following criteria from Tun-Lin et al. [36] adapted to our setting, we separately assessed houses and courtyards; for each of these, we defined a covariate with values of 1 (poor overall maintenance, garbage accumulation, and, for courtyards, overgrown vegetation) or 0 (well-maintained houses or courtyards). Finally, we noted whether routine control interventions were or were not performed in our study area in each of the last 13 months of monitoring. These interventions were carried out by municipal/state agents and military staff, and involved elimination of breeding sites, physically or with larvicides [20], [27]; while campaigns are designed to target all dwellings, interventions are effectively limited to houses whose owners are present at the time of the visits and allow control agents to inspect their property.

### Data Analyses

Our analytical approach involved two main steps. First, we used descriptive statistics, tables, and graphs to explore the data [37], and calculated naïve infestation rates (i.e., rates that assume perfect detection of vectors) of both target species for later comparison with model-based estimates (see below). Second, we implemented a set of hierarchical models of occupancy dynamics. These models explicitly account for imperfect detection, providing estimates of detection probability (denoted *p*) conditioned on occupancy (denoted ψ), and treat temporal changes in occupancy as a first-order Markov process [38]–[40]. Thus, the probability of a site being occupied in month *t* depends on the occupancy state of that site in the previous month, *t*−1. This also accounts for a form of temporal autocorrelation: when observations on the same sampling unit are positively correlated, values close in time are more similar than those separated by longer periods [40]. Apart from *p* and ψ, our occupancy dynamics models also provide information about the probability that a dwelling that is infested at time *t*−1 will become uninfested at time *t* (named local extinction probability, or ε) or that an uninfested dwelling at time *t*−1 becomes infested at time *t* (colonization probability, γ). Models can use a variety of parameterizations to represent the same occupancy-dynamic process: we chose to focus on the estimation of ψ and γ, which, combined, can yield information on ε. Parameters *p*, ψ, ε, and γ can be modeled as functions of sampling or environmental covariates. For simplicity, we model covariates on *p* and ψ.

The main assumption of the models is population closure – i.e., site-specific occupancy remains unchanged within each sampling month. In our case, closure was guaranteed by the simultaneous assessment of all traps set within each dwelling. This design could however result in nonindependence of traps set within a single dwelling: if detection in one trap increases detection probabilities in the others, this would result in some overestimation of *p* and, therefore, a negatively biased ψ estimate. Finally, the usual assumption of independence of dwellings with regard to infestation also applies; if violated, this would inflate the precision of occupancy estimates.

As summarized above, estimation of *p* requires detection histories from repeated samples or multiple traps. A ‘011’ detection history for a dwelling and month, for example, indicates that one trap was false-negative; on the other hand, all-zero histories may result from two scenarios: either the dwelling was truly uninfested or it was infested yet all traps failed to detect the vectors [38]–[40]. Since detection probability (*p*) is conditional on site-occupancy, it can be interpreted as the sensitivity of the vector-detection method – its ability to detect the presence of the vectors in dwellings that are actually infested.

Models were fit by likelihood maximization and ranked according to the Akaike information criterion corrected for small sample size (AICc) [41]. Model fitting and ranking were carried out with the freely-available software PRESENCE 4.0 [42]. To avoid repetition, further details on model specification, comparison, and selection are presented in the Results section and in Tables 1, 2, and S1.

**Table 1:** Effects of control interventions, meteorological variables, and dwelling traits on infestation rates by dengue vectors: dynamic site-occupancy models fitted to a 13-month dataset.

Species/model	ΔAICc	Covariate	β	SE	CI-lower	CI-upper
*Aedes aegypti*, 13 months						
ψ(t_max-*2-week-lag*_),γ(.),*p*(observer)	0					
		t_max-*2-week-lag*_	–0.65	0.25	–1.14	–0.16
ψ(t_max-*2-week-lag*_,control*_no-lag_*),γ(.),*p*(observer)	0.73					
		t_max-*2-week-lag*_	–0.87	0.36	–1.58	–0.16
		control*_no-lag_*	–0.81	0.62	–2.03	0.41
ψ(t_max-*2-week-lag*_,house),γ(.),*p*(observer)	2.41					
		t_max-*2-week-lag*_	–0.66	0.25	–1.16	–0.17
		house	0.22	0.66	–1.08	1.51
*Aedes albopictus*, 13 months						
ψ(t_min-*1-week-lag*_,house),γ(.),*p*(.)	0					
		t_min-*1-week-lag*_	–0.26	0.12	–0.49	–0.03
		house	0.78	0.40	–0.0005	1.56
ψ(t_min-*1-week-lag*_,control*_4-week-lag_*,house),γ(.),*p*(.)	0.87					
		t_min-*1-week-lag*_	–0.27	0.11	–0.49	–0.05
		control*_4-week-lag_*	–0.27	0.21	–0.69	0.14
		house	0.79	0.40	–0.002	1.58
ψ(t_min-*1-week-lag*_,control*_no-lag_*,house),γ(.),*p*(.)	2.51					
		t_min-*1-week-lag*_	–0.27	0.12	–0.50	–0.03
		control*_no-lag_*	0.028	0.19	–0.33	0.39
		house	0.78	0.40	–0.0002	1.56

“(.)” denotes that no covariates entered this part of the model; see text for further details. ΔAICc, variation of Akaike information criterion (corrected for small sample size) values with respect to the first-ranking model in each set; β, slope coefficient estimated for each covariate in the corresponding model; SE, standard error; CI-lower and CI-upper, limits of the 95% confidence interval; t_max-*2-week-lag*_, standardized mean of maximum daily temperatures over sampling days and the 15 days prior to sampling; t_min-*1-week-lag*_, standardized mean of daily minimum temperatures during sampling days and the previous week; house, house condition covariate; control*_no-lag_*, vector control covariate (same month); control*_4-week-lag_*, vector control covariate (previous month); observer, observer team covariate; see main text for further details on covariates.

**Table 2:** Meteorological covariate effects on dwelling infestation rates by *Aedes aegypti* and *Ae. albopictus*: dynamic site-occupancy models fitted to a 38-month dataset.

Species/model	ΔAICc	Covariate	β	SE	CI-lower	CI-upper
*Aedes aegypti*, 38 months						
ψ(t_max-*2-week-lag*_),γ(.),*p*(trap,observer)	0					
		t_max-*2-week-lag*_	–0.63	0.14	–0.90	–0.35
ψ(t_max-*1-week-lag*_),γ(.),*p*(trap,observer)	0.98					
		t_max-*1-week-lag*_	–0.57	0.12	–0.81	–0.33
ψ(r*_1-week-lag_*),γ(.),*p*(trap,observer)	6.29					
		r*_1-week-lag_*	0.50	0.14	0.23	0.77
*Aedes albopictus*, 38 months						
ψ(t_min-*1-week-lag*_),γ(.),*p*(trap)	0					
		t_min-*1-week-lag*_	–0.59	0.09	–0.77	–0.41
ψ(r*_4-week-lag_*),γ(.),*p*(trap)	21.4					
		r*_4-week-lag_*	0.46	0.09	0.28	0.64

“(.)” denotes that no covariates entered this part of the model; see text for further details. ΔAICc, variation of Akaike information criterion (corrected for small sample size) values with respect to the first-ranking model in each set; β, slope coefficient estimated for each covariate in the corresponding model; SE, standard error; CI-lower and CI-upper, limits of the 95% confidence interval; t_max-*2-week-lag*_, standardized mean of maximum daily temperatures during sampling and the previous 15 days; t_max-*1-week-lag*_, standardized mean of maximum daily temperatures during sampling days and the previous week; r*_1-week-lag_*, standardized mean of daily rainfall during sampling days and the previous week; t_min-*1-week-lag*_, standardized mean of daily minimum temperatures during sampling days and the previous week; r*_4-week-lag_*, standardized mean of daily rainfall over the month before sampling; trap, trap-type covariate; observer, observer team covariate; see main text for further details on covariates.

We fit occupancy dynamic models separately to (i) the 13-month subset of data for which we recorded vector control interventions and house/courtyard covariates and (ii) the full 38-month dataset. This resulted in a two-stage analysis. On the first stage, we focused on modeling the effects of control interventions on ψ, looking both at interventions that took place in the same month as sampling (denoted control*_no-lag_*) and during the preceding month (lagged effect, control*_4-week-lag_*). These models also consider meteorological and dwelling conditions. Since two teams were involved in vector monitoring, we also modeled detection probability (*p*) as a function of the observer team to account for possible differences in team performance [39], [40].

On the second stage, we set aside control interventions and focused on estimating time-dependent occupancy for the whole 38-month dataset, including the final 13 months of control-intervention monitoring. This second set of models also considered the effects of meteorological covariates on occupancy, albeit with a larger amount of data. Since we used two trapping devices during the first phase of monitoring, detection probabilities were modeled as a function of trap type, and, once again, as a function of the observer team. We also assessed the amount of bias present in naïve *vs.* model-derived infestation rate estimates (bias = 1 − [naïve/model-derived values]).

### Ethics Statement

Sampling was carried out with permission from dwelling owners, and did not involve endangered or protected species. SLBL holds a permanent license (27733-1) from the Brazilian Institute for the Environment and Natural Resources (IBAMA) for sampling insect vectors such as the *Aedes* species we studied.

## Results

### Descriptive Results: Observed Infestation

**Figure 3:**
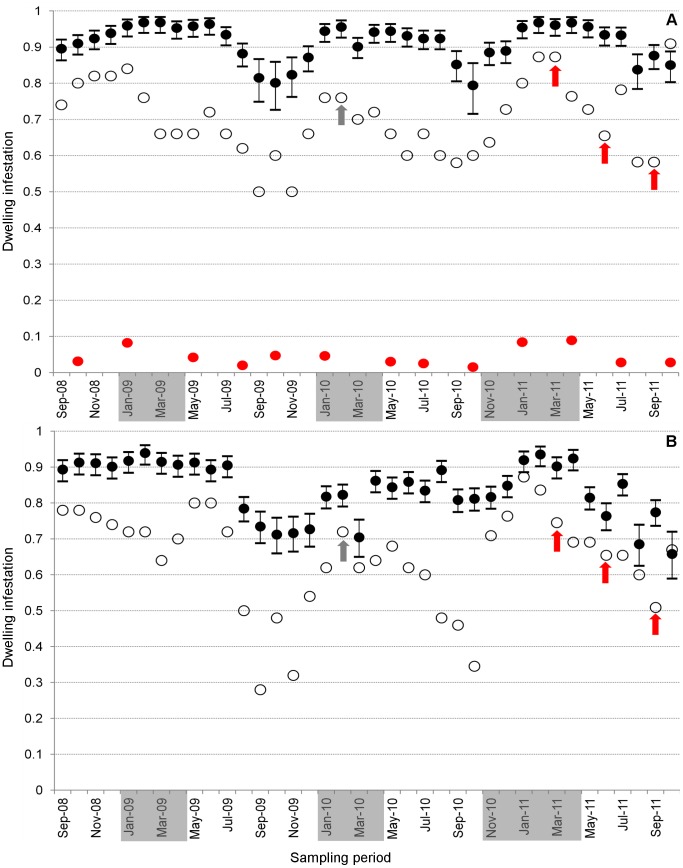
Observed and model-estimated dwelling infestation by *Aedes aegypti* (A) and *Ae. albopictus* (B). Monthly model-derived site-occupancy estimates (solid circles, with 95% confidence intervals); monthly observed infestation (empty circles); and *Ae. aegypti* infestation indices derived from 13 ‘rapid larval surveys’ [28] (red circles in panel A). On the *x* axis, grey boxes highlight the periods in which city-wide, massive *Aedes* control campaigns, called *Operação Impacto* [29], [30], took place. Arrows indicate months in which control activities were performed in our study neighborhood (red arrows, interventions included as model covariates).

**Figure 4:**
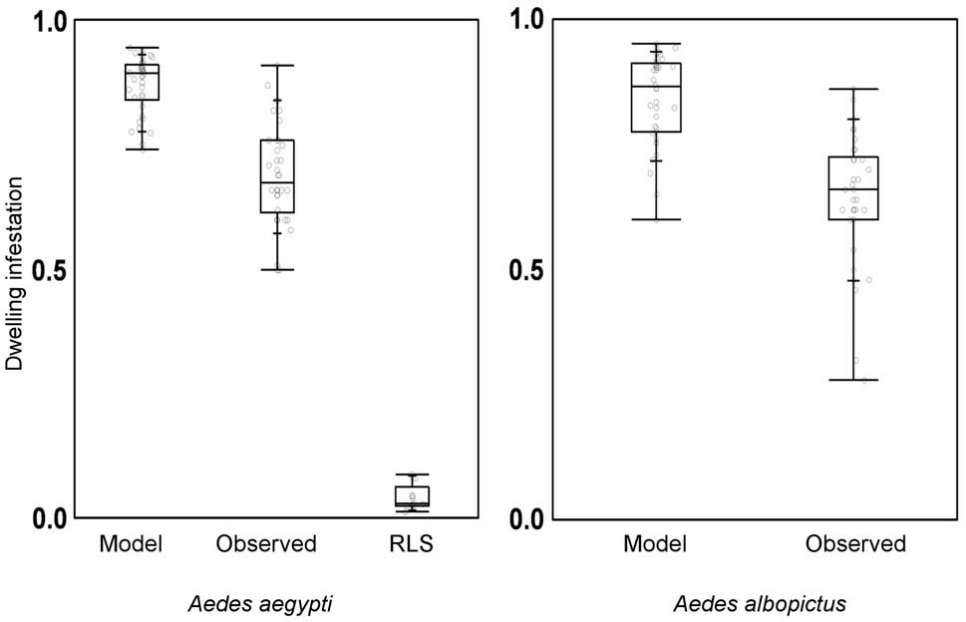
Bias in *Aedes aegypti* (left) and *Ae. albopictus* (right) observed infestation. Model-derived point estimates (“Model”) correspond to the top-ranking, 38-month dynamic model for each species; observed dwelling infestation recorded during our surveys (“Observed”) and indices of dwelling infestation by *Ae. aegypti* reported by the regular vector surveillance system, derived from ‘rapid larval surveys’ [28] (“RLS”). Monthly values (empty circles) and quartiles 50% (horizontal line within box), 25%–75% (box lower-upper limits), 10%–90% (short lines), and 0%–100% (bottom-top lines) are shown. All values are presented as proportions.

Both vector species were detected in a high proportion of dwellings throughout the study period (Figure 2), with harmonic mean values of 0.68 for *Ae. aegypti* (range, 0.50–0.91) and 0.61 for *Ae. albopictus* (range, 0.28–0.86). There was an apparent relationship between site-occupancy and weather (Figures 2 and 3). The particularly hot and dry period of June-September 2009 coincided with a sharp decrease of *Ae. albopictus* infestation: observed values fell from ∼0.70–0.80 to ∼0.30–0.50. A less marked decline was also apparent for *Ae. aegypti*. Both species, however, quickly recovered with the onset of the rainy season. Dwelling infestation indices (the World Health Organization ‘house index’) reported by routine municipal surveillance for our study neighborhood, based on 13 ‘rapid larval surveys’ [28] carried out between October 2008 and October 2011 (Figures 3 and 4), yielded a harmonic mean of just 0.033 (range, 0.015–0.089). These descriptive results rely on the assumption that vectors were absent from sites where they were not observed; however, no perfect vector-detection method is available. The modeling results summarized in the next section address this key limitation.

### Modeling Results I: Effects of Control Interventions

On the first stage of our analysis we modeled the effects of vector control interventions carried out by local health authorities on site-occupancy by *Ae. aegypti* and *Ae. albopictus*. These models used data from 55 dwellings monitored from October 2010 to October 2011 with up to three ovitraps per dwelling and month. Overall, the data encompass results from 1907 ovitraps, of which 849 detected *Ae. aegypti* and 828 detected *Ae. albopictus*.

*Aedes aegypti* detection/non-detection data are best explained by a model with just one covariate on ψ, the average of maximum daily temperatures measured with a 2-week-lag (t_max-*2-week-lag*_), which had a negative effect on site-occupancy (Table 1). The second-ranking model is also substantially supported by the data (ΔAICc = 0.73); it includes the additive effects of t_max-*2-week-lag*_ and control interventions carried out during the same month (control*_no-lag_*) on ψ. The effect of temperature was again negative; this model also yielded a negative point estimate of the control coefficient, but uncertainty about this estimate is large and the 95% confidence interval overlaps zero (Table 1). Among candidate models including dwelling covariates, the one with the lowest AICc estimates a weak, positive effect of poor house condition on infestation, but, again, the estimate of this effect is too uncertain to draw any strong conclusions (Table 1).

The top-ranking model for *Ae. albopictus* estimates a negative effect of 1-week-lagged minimum temperatures (t_min-*1-week-lag*_) on infestation; in addition, the model suggests that houses in poor condition might have been at a slightly higher risk of infestation, albeit the estimated coefficient’s 95% confidence interval includes zero (Table 1). Adding control interventions carried out the month before (control*_4-week-lag_*) resulted in a model that fits reasonably well (ΔAICc<1). For this second model, the negative coefficient of control*_4-week-lag_* on ψ is nevertheless small and imprecise, with confidence intervals including zero (Table 1). Finally, a model with t_min-*1-week-lag*_, house condition, and control*_no-lag_* as covariates also performed reasonably well; it estimated a small positive coefficient, again not different from zero, of control interventions (Table 1). The null model (without any covariates) and those models exploring the effects of courtyard covariates all had ΔAICc≥3 (see Table S1).

### Modeling Results II: Long-term Site-occupancy Dynamics

The results in the previous section show that modeling time-specific occupancy as a function of control interventions or dwelling-level covariates did not improve the ranking of the models. Therefore, we felt justified to extend modeling to the full dataset – focusing on the potential effects of meteorological variables on site-occupancy by *Aedes* species. The analyses make use of a 38-month dataset including individual results of 5799 trap-weeks, which detected *Ae. aegypti* on 2641 occasions and *Ae. albopictus* on 2538 occasions. In these models, colonization probability (denoted γ) was constrained to be constant across months, while monthly local (dwelling-level) extinction probabilities (ε), of primary interest in the context of vector control, were derived from ψ and γ estimates as described in MacKenzie et al. [39], [40].

The full *Aedes aegypti* data were best explained by a model including the 2-week-lagged average of daily maximum temperatures (t_max-*2-week-lag*_), which had a negative effect on site-occupancy probabilities (Table 2). The model with t_max-*1-week-lag*_ as a covariate on ψ also fitted the data well, and estimated a similar effect to that of t_max-*2-week-lag*_ (Table 2). The remaining models that we examined, including a null model without any covariates, performed substantially worse than these two top-ranking models (see Table S1). Among models that included rainfall covariates, the best-performing one had a ΔAICc = 6.29 and estimated a positive effect of total rainfall (r*_1-week-lag_*) on ψ (Table 2).

**Figure 5:**
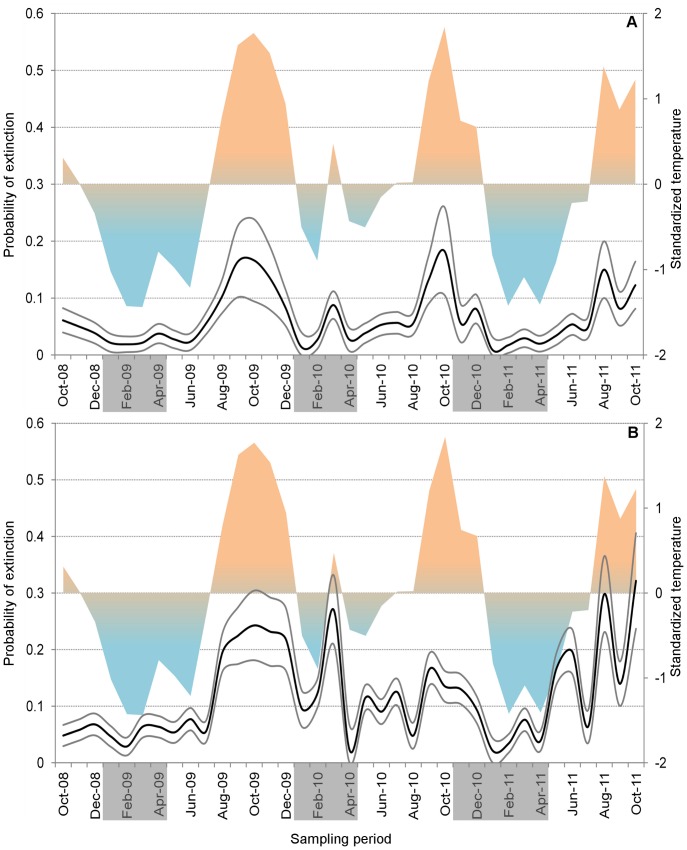
Derived estimates of local extinction probabilities (ε) for *Aedes aegypti* (A) and *Ae. albopictus* (B). For each species, ε estimates (bold black lines) and 95% confidence intervals (thin grey lines) were derived from the best-performing (lowest AICc) 38-month model. We also plot variation (*z*-scores) of average maximum temperatures during sampling days and the previous two weeks (t_max*-2-week-lag*_, right *y* axes in each panel; colored areas); this was the meteorological covariate in the best *Aedes aegypti* model. On the *x* axis, grey boxes highlight the periods in which city-wide, massive *Aedes* control campaigns, called *Operação Impacto* [29], [30], took place; note that they coincide with months of very low ε values.

Figure 3A shows monthly site-occupancy estimates for *Ae. aegypti* derived from the lowest-AICc model. With few exceptions, point estimates were consistently >90% (harmonic mean 0.91; range, 0.79–0.97) and showed a weak seasonal pattern (Figure 3A). Model-based infestation estimates are about 30% higher than observed values based on ovitrap results (median bias, 0.29) (Figure 4). The estimated average sensitivity of ovitraps at detecting infestation by *Ae. aegypti* varied from *p* = 0.48 (95% confidence interval 0.45–0.51) to *p* = 0.65 (0.63–0.67), depending on which field team performed monitoring (details not shown); 1355 out of 4553 ovitrap-weeks and 450 out of 1246 Adultrap-weeks did not detect *Ae. aegypti* in dwellings where other traps yielded evidence of infestation. Local extinction probability estimates were overall very low (harmonic mean ε = 0.04; range, 0.01–0.18), reaching higher values in hotter months (Figure 5A); mean site-colonization probabilities were estimated as γ = 0.66 (95% confidence interval 0.54–0.76) over the study period.

The best-ranking *Ae. albopictus* model included only one site-occupancy covariate, t_min-*1-week-lag*_, which had a negative effect on ψ (Table 2). The remaining models performed substantially worse (ΔAICc>20), but several of the candidate specifications we tested had convergence problems. The only model with a rain covariate estimates a positive effect of 4-week-lagged rainfall on site-occupancy by *Ae. albopictus* (Table 2). Site-occupancy estimates derived from the best-ranking model are presented in Figure 3B. As with *Ae. aegypti*, monthly ψ values were always high (harmonic mean 0.83; range, 0.66–0.94), with minimum ψ = 0.66 (95% confidence interval 0.59–0.72) in October 2011. Monthly *Ae. albopictus* ψ estimates were more unstable than those of *Ae. aegypti*, with relatively strong fluctuations after the dry-hot summer of 2009 (Figure 3B). Observed infestation (based on ovitraps) was also biased downwards (by ∼26%) in our *Ae. albopictus* data (Figure 4), yet ovitraps were fairly sensitive at detecting *Ae. albopictus* (*p* = 0.63, 95% confidence interval 0.62–0.65). Monthly local extinction probabilities were low: harmonic mean ε = 0.07, range 0.02–0.32, with the maximum value estimated for October 2011 (Figure 5B). Mean dwelling colonization probability was estimated as γ = 0.59 (95% confidence interval 0.51–0.66).

## Discussion

Reliable dwelling infestation estimates are critical for decision-making in the context of dengue vector surveillance and control. The definition of programmatic goals, the management of resources, and the assessment of intervention effects all rely heavily upon such estimates. Using a large dataset and a sound analytical approach we have shown that routine vector surveillance and control are both performing poorly: at least in our study setting, (i) ‘rapid larval surveys’ yielded dwelling infestation indices that were markedly lower than the site-occupancy rates based on ovitrap data, and (ii) control campaigns had negligible effects on site-occupancy. Our results suggest that combining ovitrap-based surveillance (e.g., [43]–[45]) with analytical methods that account for imperfect detection (e.g., [21], [22], [38]–[40]) would help quantitatively assess, and likely enhance, dengue control programs. Moreover, from a disease transmission perspective, the presence of foraging gravid females in a dwelling, which ovitraps detect with reasonable sensitivity, is arguably more important than the presence of larvae, which is what ‘rapid larval surveys’ aim to detect.

Before discussing our findings any further, we identify several study limitations to keep in mind when interpreting the present results. First, we used detection/non-detection data, ignoring variations in vector abundance. However, presence-absence and abundance data seem to correlate well (e.g., refs. [46], [47]), and, importantly, both empirical and modeling results suggest that *Ae. aegypti* abundance thresholds (above which dengue transmission is maintained) are typically very low, i.e., ∼0.5–1 female per person or ∼0.5–1.5 pupae per person [48], [49]; therefore, the probability that at least one gravid female is present in a dwelling, which we modeled here, is clearly an epidemiologically relevant parameter. Second, some of our data may violate the assumption of independence of traps with regard to detection and of dwellings with regard to infestation; this may result in negatively biased ψ estimates with overly narrow confidence intervals. The high ψ estimates we found suggest that this problem was, in practice, negligible – our conclusions would not change because of somewhat broader confidence intervals. In addition, we measured only, and coarsely, a small number of covariates, but these were selected because of their known importance for our target species (e.g., [33]–[36], [50]). Our ‘control’ covariate included control interventions in just three out of 13 months of assessment, and this clearly lowered the precision of effect-size estimates: it seems possible that with more intervention data we might be able to detect a small effect whose 95% confidence interval could exclude zero. Yet, since ∼70–90% of dwellings remained infested despite control interventions, ‘statistical significance’ would in this case be of no practical importance [49]. Acknowledging these caveats, we feel confident that our models adequately estimate infestation rates as well as some of the major determinants of those rates in our study area. The main difference between our approach and previous attempts to assess infestation by dengue vectors is that we go beyond measuring indirect indices of infestation (i.e., adult or larvae presence/absence or counts) to produce statistical estimates of the probability that our study units (dwellings) are occupied by the target vector species.

We found little evidence that dwelling infestation rates decreased measurably as a result of the vector control campaigns carried out by local health authorities in our study neighborhood. These campaigns involved the elimination/treatment of thousands of artificial breeding containers [29], [30], and were expected to have larger effects on *Ae. aegypti*, which unlike *Ae. albopictus* rarely breeds in natural water collections [5], [17]. Our results show, however, no measurable effect of control interventions on any of the two vectors (Table 1); indeed, females of both species consistently continued to lay eggs, and probably forage, in most of the dwellings we surveyed, irrespective of whether control interventions had or had not taken place in the neighborhood. Our models suggest that this lack of effect could be related to the fact that interventions are usually planned to coincide with the wet-cool season, which is when local extinction probabilities drop to their lowest values (Figure 5). Summer interventions might perhaps be more effective [51], since they could synergize the negative effects of high temperatures on *Ae. albopictus* and *Ae. aegypti* detected by our models and in previous studies (e.g., [50]–[53]). These negative effects of high temperature, however, have to be considered in the particular context of our study. First, the extrinsic incubation period of dengue virus and the vector’s gonotrophic cycle can both be shortened by warmer weather, increasing transmission risk; second, relatively high temperatures probably favor vector development in overall cooler climates [10].

One practical implication of our findings is that *Aedes* breeding sites appear to be often overlooked by vector control agents during active surveillance and, principally, in control campaigns. This suggests a key drawback to be addressed in the development of novel *Aedes* control strategies, which should not heavily depend on the ability of control agents to detect breeding sites while inspecting premises. Two major candidate strategies address this problem from very different, but complementary, perspectives: (i) the use of adult mosquitoes to transfer potent larvicidal particles from contaminated ‘dissemination stations’ to clean breeding sites [54], and (ii) the release of mosquitoes carrying transgenes [55], [56] or specific *Wolbachia* strains [57] that impair reproduction and/or reduce competence to transmit dengue virus.

### Conclusions

The reported bias of infestation indices that do not account for imperfect detection suggests that the findings of most dengue vector ecology studies must be interpreted with caution. Even ovitraps, which performed relatively well, yielded naïve infestation rates that were consistently biased downwards by about 30%. The analytical strategy we used here incorporates this sampling-process uncertainty, and could therefore substantially contribute to this field of inquiry.

Finally, our results suggest two promising avenues for the much-needed improvement of dengue vector surveillance [58]. First, simple hay infusion-baited ovitraps [32] should be preferred to ‘rapid larval surveys’: they are more sensitive and provide a measure of dwelling infestation by foraging gravid females (see also, e.g., refs. [43]–[45], [59]). Second, the repeated-sampling approach we used considerably improves infestation rate estimates by explicitly taking imperfect detection into account. Enhanced entomological surveillance systems and data analyses that explicitly account for the detection process would, in turn, allow for reliably assessing the effects of control interventions, irrespective of the specific tactics employed. Without such an assessment, the grounds on which massive public spending is directed towards dengue vector control (e.g., [60]) remain questionable.

## Supporting Information

Table S1 **The complete sets of site-occupancy dynamic models.**
(XLSX)
